# Improving Traps for Spotted Lanternflies, *Lycorma delicatula* (Hemiptera: Fulgoridae), by Leveraging Their Own Signals

**DOI:** 10.3390/insects16090930

**Published:** 2025-09-04

**Authors:** Miriam F. Cooperband, Kelly M. Murman

**Affiliations:** Forest Pest Methods Laboratory, USDA–APHIS–PPQ, 1398 W. Truck Rd., Buzzards Bay, MA 02542, USA

**Keywords:** semiochemicals, insect communication, live bait, traps, lures, honeydew, body volatiles, pheromones, substrate vibrations, aggregation

## Abstract

The spotted lanternfly, *Lycorma delicatula* (Hemiptera: Fulgoridae) (SLF), is a damaging invasive pest and generalist phloem feeder that has been found in 18 states in the United States. It has a complex multimodal communication system involving semiochemicals, emitted both from their honeydew and their bodies, and substrate-borne vibrations. Sensitive and effective traps for detection and survey are essential management tools, but no potent lures for SLF exist yet. We sought to test an alternative that relies on live-trapped SLF acting as lures to improve trap efficacy after the first SLF is captured. SLF circle traps were modified by replacing the commonly used plastic collection bag with a mesh bag pinned to the tree trunk. These allowed the trapped SLF to remain alive and generate signals through the mesh bag, thus leveraging their natural modes of communication to draw additional SLF into the traps. We compared mesh and plastic bags over three years targeting fourth instars and adults and found that prior to oviposition, circle traps with mesh bags captured significantly more fourth instar (70% mesh: 30% plastic) and adult SLF (59% mesh: 41% plastic) compared to plastic bags, but during oviposition time, the results were mixed.

## Simple Summary

Spotted lanternflies, *Lycorma delicatula* (Hemiptera: Fulgoridae) (SLF), have a complex multimodal communication system involving semiochemicals, emitted both from their honeydew and their bodies, and substrate-borne vibrations. However, potent lures for SLF have not yet been developed. We sought to test an alternative that relies on live-trapped SLF acting as lures to improve trap efficacy after the first SLF is captured. Circle traps were modified by replacing the commonly used plastic collection bag with a mesh bag pinned to the tree trunk, allowing trapped SLF to feed, excrete honeydew, emit body volatiles, and generate substrate vibrations through the mesh bag, thus leveraging their natural modes of communication to draw additional SLF into traps. Three years of results targeting fourth instars and adults show that prior to oviposition, circle traps with mesh bags significantly improved trap capture for fourth instar and adult SLFs compared to plastic bags, but during oviposition time, the results were mixed.

## 1. Introduction

The spotted lanternfly (SLF), *Lycorma delicatula* (White) (Hemiptera: Fulgoridae), is an invasive pest with piercing sucking mouthparts that feeds heavily on the vascular systems of a wide range of host plants [1,2,3,4]. The host range, damage, and the limited tools for survey and control of SLF have been reviewed [5,6,7,8,9,10,11,12,13,14,15,16,17,18,19,20,21,22,23,24]. Early in its introduction, trapping relied on sticky tree bands which successfully trapped smaller nymphs, but larger instars and adults evaded capture, and vertebrate by-catch and trap saturation were problematic [15,25,26,27]. Inverting sticky tree bands helped to alleviate some of these problems [28], but further trap design improvements have since been implemented [29,30,31]. Currently, the most efficient SLF traps available are circle trunk traps which funnel upward-walking SLF into a clear plastic bag, an improvement from the same traps fitted with a catch jar [28,30]. The only lure that has been found to improve trap capture, so far, is a high-release methyl salicylate lure, but these have produced mixed results in field trials [25,29,30,32]. In electrophysiological and laboratory behavioral studies, methyl salicylate consistently outperformed most other candidate compounds. In comparison to other honeydew and plant compounds, methyl salicylate elicited some of the largest electrophysiological responses from both SLF antennae [33] and apical sensilla on the proboscis, which were found to be more sensitive to methyl salicylate than most other compounds by an order of magnitude [34]. Methyl salicylate was also the most attractive of 17 antennally-active individual host plant volatiles tested in behavioral bioassays [35] and was found to be attractive to both nymphs and adults [25]. However, methyl salicylate lures were not able to outcompete other factors in the environment such as larger tree size or pre-existing SLF aggregations [32]. Efforts to develop a more potent lure containing attractive blends have been unsuccessful so far (MFC, unpublished) [36].

Recent studies have found that SLF form aggregations using a multimodal communication system incorporating semiochemicals emitted from their bodies [37,38] and honeydew [39], as well as substrate vibrations, which we are currently working to describe (MFC, unpublished) [36,40,41]. Volatile chemical profiles from SLF honeydew and bodies have been described, and males have been shown to orient to these volatiles in laboratory and field bioassays [36,38,39], particularly before and during mating time. However, due to the large number of antennally-active components [33], a potent semiochemical lure has not yet been developed to attract SLF for aggregation or mating. Further complicating matters, we have recorded SLF communicating through substrate vibrations during courtship and are in the process of describing their calls [41], but the role substrate vibrations play in aggregation and mate location by SLF is not fully understood.

Thus, there is ample evidence indicating that SLF communicate both chemically and through substrate vibrations in order to aggregate and locate mates. However, without yet knowing the specific chemical or vibratory components required for potent attraction, we currently can only utilize the insects’ natural signals. Other insect species that have chemical or multimodal communication have been successfully trapped using live insects as the lure [42,43,44,45,46,47,48,49,50]. Here, we sought to find a practical way of employing the natural multimodal signals generated by SLF as a lure in order to improve trap capture rates until a synthetic lure can be developed. One way to convert captured SLF into a lure is by allowing them to continue to live, feed, and broadcast signals to their conspecifics after being captured through a simple circle trap modification which replaces the plastic collection bag with a mesh collection bag that is attached to the tree trunk.

## 2. Materials and Methods

### 2.1. Field Sites and Timing

Three field studies were conducted in 2022, 2023, and 2024 to explore and evaluate the potential for using captured SLF as a lure to attract additional SLF into traps. In 2022, the first such exploratory experiment was conducted between 2 August and 25 October at field sites in Sussex County, New Jersey, with 13 replicated blocks. In 2023, the experiment was conducted again from 24 July to 23 October at field sites in Sussex and Warren Counties, New Jersey and Monroe County, Pennsylvania with 14 replicated blocks. In 2024, five volunteers from USDA APHIS PPQ Field Operations were enlisted to set and service traps in latitudes farther south, where SLF had more recently invaded. This third experiment was conducted between 6 July and 6 November, at field sites in New Castle County in Delaware (N = 10); Caroline, Hanover, and Roanoke Counties in Virginia (N = 6); and Brooke, Jefferson, and Taylor Counties in West Virginia (N = 10), for a total of 26 replicated blocks (Supplementary Table S1).

Trapping studies were conducted on a variety of forested private residential and public properties on mature *Ailanthus altissima* (Mill.) Swingle (Sapindales: Simaroubaceae) trees, the preferred host of SLF. Trees ranged from 14.5 cm to 33.8 cm diameter at breast height (DBH), but the DBH of any two trees in a single block did not differ more than 5 cm. Each block consisted of either three (2022 and 2023) or two (2024) trees, spaced 3–7 m apart either in a triangle or a line. Blocks were at least 15 m away from other blocks.

Every tree in each block received a circle trunk trap for spotted lanternfly (detailed in Section 2.2 below), placed at breast height with the opening and trap bag oriented approximately on the north side of the tree. Traps were serviced approximately every two weeks by removing and replacing the trap bag containing the captured SLF, which were then frozen, and later sorted and counted. At the time traps were serviced, the treatments assigned to each tree were rotated to control for variation or differences between the trees in each block. The 2022 and 2023 experiments were pilot studies, and the third tree received an experimental trap bag that was omitted from analysis. In some cases, a trap was found to be damaged by wind or animals, in which case all trees in the affected block were excluded from analysis for that trapping period. Dates of trapping periods, stages, and numbers of blocks are provided in Table 1. In 2024, because the five volunteers in different states each had different trap servicing schedules, with variation in the length, timing, and number of trapping periods, we grouped data into date ranges when traps were serviced, which resulted in some blocks being sampled more or fewer times in the 2024 trapping period groupings.

Trapping periods are reported according to their predominant SLF developmental stages as previously defined: 4th instar, Early-1 (which starts when adults emerge), Early-2 (which starts when an abrupt shift towards a female-biased sex ratio is observed on some trees), Mid (which starts with the first observation of mating in the field), Late-1 (which starts with the first egg mass in the field), Late-2 (which starts two weeks after Late-1), and Late-3 (which starts two weeks after Late-2) (Table 1).

### 2.2. Trap Description

**Figure 1:**
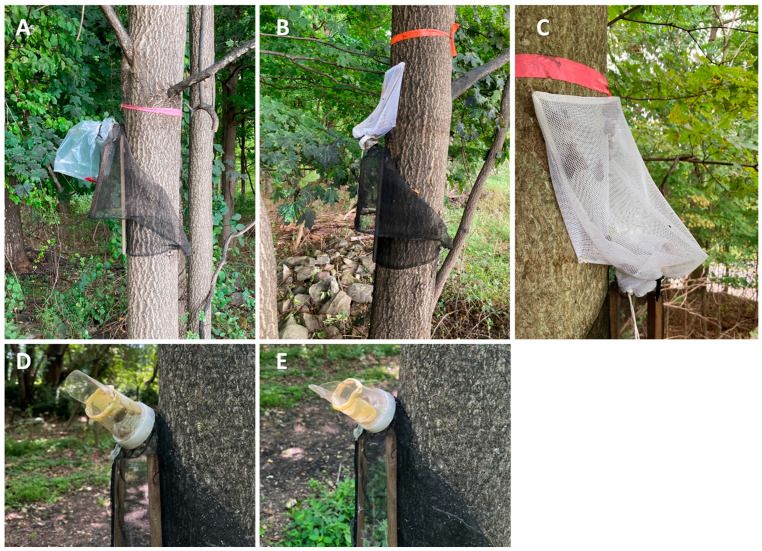
The circle trunk trap designed to capture spotted lanternflies, *L. delicatula* (SLF), fitted with (**A**) a plastic collection bag or (**B**) a mesh collection bag. A close-up of a mesh collection bag (**C**) shows how it was pinned to the tree trunk to allow the captured SLF to feed through the mesh. The orientation of the plastic tab that keeps the collection bag opening clear is detailed for plastic (**D**) and mesh (**E**) collection bag attachments.

To test whether SLF could be kept alive in traps to function as a lure, standard spotted lanternfly circle traps (GL/GL-4011-00, Great Lakes IPM™, Vestaburg, MI, USA) were used to compare the commonly used plastic collection bag, in which SLF typically die shortly after capture (Figure 1A), with an experimental mesh collection bag that we attached to the tree to allow captured SLF to live, feed, and emit signals (Figure 1B). In 2022 and 2023, but not 2024, each plastic collection bag (20.3 × 10.1 × 38.1 cm Mil Gusseted Poly Bags, S-5412, Uline, Allentown, PA, USA) received an insecticidal strip (Vapona II, 2,2-dichlorovinyl dimethyl phosphate (10%), Hercon Environmental, Emigsville, PA, USA). These strips were added to prevent captured insects from escaping, and to prevent trapped predators from eating the data. However, in 2024, kill strips were omitted because they did not significantly improve capture rates [28,30]. Mesh bags were tested in 2022 and 2023, with two sizes of mesh openings, respectively (28 × 38 cm Frienda nylon drawstring laundry bags with 2 mm × 2 mm openings, Shenzhen Chenying Network Technology Co., Ltd., Shenzhen, China; and 30 cm × 46 cm MB18 nylon equipment bag with 4 mm × 4 mm openings, Champion Sports, Marlboro, NJ, USA). In 2024, with double the number of field blocks and a longer field season, washed bags from the prior two years were reused, and additional bags with the larger openings were purchased, thus 61 and 103 bags with the smaller and larger mesh sizes, respectively, were used in 2024.

To allow captured SLF to feed, the tree-facing surface of the mesh bag was attached flush to the tree trunk above the trap entrance with the outward-facing side of the bag loose enough to allow the SLF space to move around. Five push pins were used inside each mesh bag to pin it in place on the tree (Figure 1C). As with plastic bags, the mesh bag opening was placed over the trap entrance and cinched tight with a zip tie. The plastic tab that extends from the trap entrance to keep the opening of the bag clear was positioned nearest the tree for plastic bags, and away from the tree for mesh bags (Figure 1D,E). In both cases, the position of the tab served to hold the bag open to allow SLF to enter. Thus, captured SLF could enter and move around inside the mesh collection bag, contact the trunk, feed through the attached mesh, generate honeydew, emit body volatiles, and produce substrate vibrations. The intent was for these signals to attract SLF from outside the trap, essentially using the captured SLF as living lures. We targeted late 4th instars and adult SLF, which are capable of feeding through the bark on the tree trunks.

To quantify the extent that SLF would feed through mesh bags attached to the trunk and remain alive longer than in plastic bags, in 2022, at the end of two 2-week trapping periods ending on 30 August and 27 September, and prior to trap servicing, SLF were observed through the bags and the number of SLF that appeared to be feeding through the mesh, and the number that were still alive and moving around, were recorded. After that, the SLF bags were collected and frozen, and the total SLF in each bag were counted. The number of live SLF was subtracted by the total number of SLF to determine the number of dead SLF.

### 2.3. Statistical Analysis

At the end of each trapping period, the captured SLF were frozen and counted, and the sex of adults was determined and quantified, except in 2022 when Mid to Late-1 SLF were counted in the field so their sex was not determined. After trap servicing, the numbers of 3rd instars, 4th instars, adult males, adult females, total adults (this included damaged adults that could not be sexed), and total SLF captured per day per trap in each trapping period were quantified. Datasets were evaluated and found to have nonparametric distribution. Therefore, matched-paired (blocked) data were analyzed using a Wilcoxon rank sums 2-sample test, with α = 0.05 (JMP v15.2.0, SAS Institute Inc., Cary, NC, USA). Treatments were compared for each trapping period to determine whether there was a significant preference for one treatment or the other. The percent of SLF captured in each treatment, and the total number (n) captured in each trapping period, are reported. Mesh and plastic matched-paired trap totals over all three years, and all rounds, were compared for 4th instars, and for males and females before and after oviposition started (21 September), using Wilcoxon rank sums 2-sample test with α = 0.05, because log transformation was unsuccessful at normalizing data.

## 3. Results

In 2022 and 2023, Early-1 started on 27 and 31 July, Early-2 started on 29 and 28 August, Mid started on 12 and 11 September, and Late-1 started on 21 and 20 September, respectively. In 2024, volunteers reported the start of Early-1 between 24 June and 15 July (five observations), the start of Early-2 between 22 and 27 August (two observations), the start of Mid on 4 September (one observation), and the start of Late-1 between 4 and 17 September (three observations).

Observations through bags were made prior to trap servicing on 30 August and 27 September in 2022 to characterize the frequency of SLF that were alive and that appeared to be feeding through the mesh (N = 26). In all mesh bags, 29% of 4th instars (n = 14) and 45% of adults (n = 1558) were alive and with one 4th instar (7%) and 375 adults (24%) that appeared to be feeding through the mesh. Conversely, one 4th instar (6%, n = 17) and six adults (1%, n = 521) were alive in all plastic bags (Table 2).

**Figure 2:**
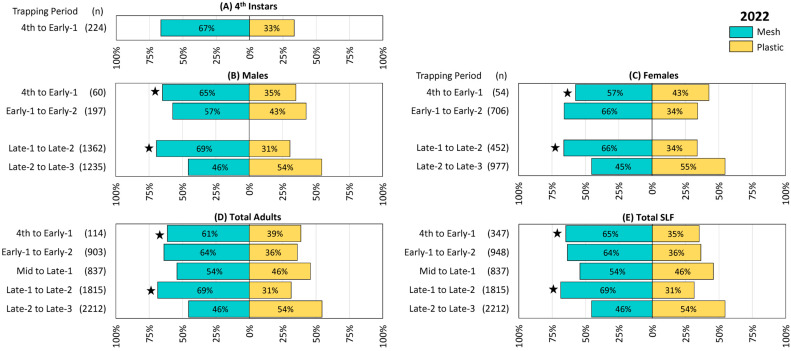
The percentage and number (n) of spotted lanternflies (SLF), *L. delicatula*, captured in each treatment and time period in 2022 (in New Jersey), using circle traps with either mesh or plastic collection bags. (**A**) Fourth instar SLF, (**B**) adult male SLF, (**C**) adult female SLF, (**D**) total adult SLF (including those that could not be sexed), and (**E**) total SLF. Every trapping period, collection bags were rotated among paired circle traps in 13 blocks. Stars indicate the ranks of SLF trapped in paired mesh and plastic traps were significantly different (Wilcoxon 2-sample test, N = 13, *p* < 0.05).

**Figure 3:**
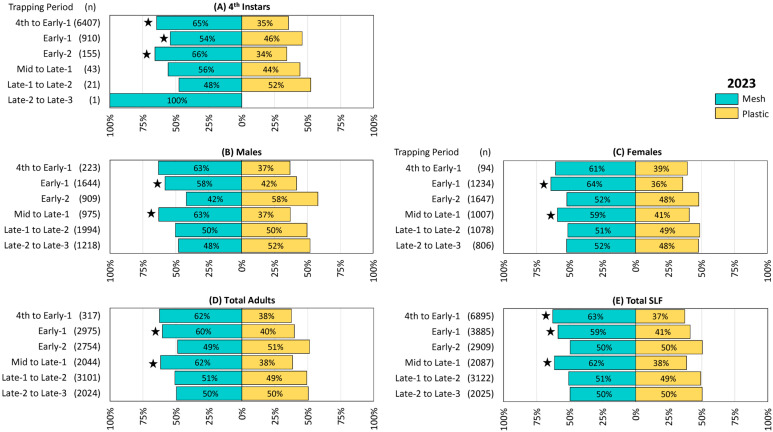
The percentage and number (n) of spotted lanternflies (SLF), *L. delicatula,* captured in 2023 in 14 pairs of circle traps, with either mesh or plastic trap collection bags rotated over 2-week trapping periods in New Jersey and northeastern Pennsylvania. (**A**) Fourth instars, (**B**) adult males, (**C**) adult females, (**D**) total adults, and (**E**) total SLF captured. Stars indicate the ranks of SLF trapped in paired mesh and plastic traps were significantly different (Wilcoxon 2-sample test, N = 14, *p* < 0.05).

**Figure 4:**
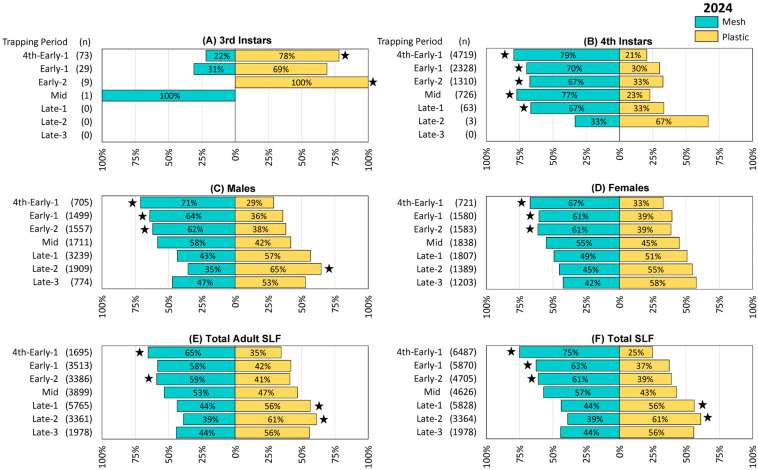
The percentage and number (n) of spotted lanternflies (SLF), *L. delicatula*, captured in each treatment and time period in 2024 (in Delaware, West Virginia, and Virginia, combined), using circle traps with either mesh or plastic collection bags. (**A**) Third instar SLF, (**B**) 4th instar SLF, (**C**) adult male SLF, (**D**) adult female SLF, (**E**) total adult SLF (including those that could not be sexed), (**F**) total SLF. Stars indicate the ranks of SLF trapped per day in paired mesh and plastic traps were significantly different (Wilcoxon 2-sample test, N = 26, *p* < 0.05).

The trapping results from 2022, 2023, and 2024 are illustrated in Figure 2, Figure 3 and Figure 4, respectively. The total number of SLF captured were 5605, 20,923, and 32,860 in 2022, 2023, and 2024, respectively. Remarkably, in each of the three years 57% of the total SLF were captured by the mesh bags, and 43% by the plastic bags.

In 2022, numerically more 4th instar SLF were captured in mesh bags than in plastic, but the difference was not significant (Figure 2A). Numerically more males (Figure 2B) and females (Figure 2C) were also caught during all but the last trapping period, but with differences being significant only during the first and fourth trapping periods (4th to Early-1, males Z = −3.57, *p* < 0.001, females Z = −2.66, *p *= 0.008; and Late-1 to Late-2, males Z = −4.11, *p* < 0.001, females Z = −3.59, *p* < 0.001), and adults were not sexed during the third trapping period. Results for total adults (Figure 2D) and total SLF (Figure 2E) included those that were not sexed, and again were numerically greater for mesh than for plastic, with significant preferences for mesh only in the first and fourth trapping periods (4th to Early-1 adults, Z = −3.97, *p* < 0.001, total, Z = −2.19, *p* = 0.028; Late-1 to Late-2 adults, Z = −4.00, *p* < 0.001, total Z = −4.00, *p* < 0.001). The only trapping period where numerically more SLF were captured in plastic than in mesh bags was Late-2 to Late-3, but this and all remaining trapping periods in this study did not differ significantly.

Trapping results from 2023 were similar to those from 2022. Numerically more 4th instar SLF were captured in mesh bags in all six trapping periods except for the fifth (Late-1 to Late-2), and differences were significant in the first three trapping periods (4th to Early-1 Z = −2.26, *p* = 0.024, Early-1 Z = −2.04, *p* = 0.041, and Early-2 Z = −1.98, *p* = 0.048) when greater numbers were captured (Figure 3A). Mesh bags captured numerically more males in three trapping periods, 4th to Early-1, Early-1, and Mid to Late-1, with the latter two being significantly different (Early-1 males Z = −2.46, *p* = 0.014, Mid to Late-1 males Z = −2.87, *p* = 0.004) (Figure 3B). Numerically more females were captured in mesh bags in all six trapping periods, with significant differences in Early-1 (Z = −3.15, *p* = 0.002) and Mid to Late-1 (Z = −2.83, *p* = 0.005) (Figure 3C). Total adults, which included any adults whose sex could not be determined, were numerically higher in mesh bags over all trapping periods except for Early-2 and Late-2 to Late-3, with significant differences in Early-1 (Z = −2.78, *p* = 0.005) and Mid to Late-1 (Z = −3.06, *p* = 0.002) (Figure 3D). Total SLF were numerically greater in mesh bags than in plastic bags over all trapping periods except for Early-2 and Late-2 to Late-3, with differences being significant in 4th to Early-1 (Z = −2.05, *p* = 0.040), Early-1 (Z = −2.87, *p* < 0.004), and Mid to Late-1 (Z = −3.19, *p* = 0.001) (Figure 3E). The remaining comparisons in this study did not differ significantly.

Trapping results from 2024 were again similar to those from the preceding two years, with the exception that 3rd instar SLF were also captured and quantified. Numerically more 3rd instars were captured in plastic bags in the first three trapping periods, 4th to Early-1, Early-1, and Early-2, with significant differences in the first (4th to Early-1 Z = 3.44, *p* < 0.001) and third trapping periods (Early-2 Z = 2.40, *p* = 0.016) (Figure 4A). Significantly more 4th instar SLF were captured in mesh bags in the first five trapping periods (4th to Early-1 Z = −3.98, *p* < 0.001, Early-1 Z = −2.67, *p* < 0.008, Early-2 Z = −2.77, *p* = 0.006, Mid Z = −3.13, *p* = 0.002, Late-1 Z = −2.60, *p* = 0.009) (Figure 4B). Mesh bags captured numerically more males through Mid and plastic bags captured numerically more males from Late-1 onward, with significant differences in the first three trapping periods and during Late-2 (4th to Early-1, Z = −2.16, *p* = 0.014; Early-1, Z = −3.27, *p* = 0.001; Early-2, Z = −2.09, *p* = 0.037; Late-2, Z = 2.36, *p* = 0.019) (Figure 4C). Numerically more females were captured in mesh bags through Mid as well, after which plastic caught numerically more females, with significant differences in the first three trapping periods (4th to Early-1, Z = −3.53, *p* < 0.001; Early-1, Z = −2.63, *p* = 0.009; Early-2, Z = −2.914, *p* = 0.004) (Figure 4D). Similarly, with the inclusion of adults whose sex could not be determined, total adults caught were numerically higher in mesh bags than in plastic through Mid, and higher in plastic bags from Late-1 onward, with significant differences in 4th-Early-1 (Z = −3.45, *p* < 0.001) and Early-2 (Z = −2.86, *p* = 0.004), Late-1 (Z = 2.07, *p* = 0.039), and Late-2 (Z = 2.65, *p* = 0.08) (Figure 4E). Likewise, total SLF were numerically higher in mesh bags than in plastic bags through Mid and higher in plastic from Late-1 onward, with significant differences in 4th to Early-1 (Z = −4.33, *p* < 0.001), Early-1 (Z = −2.72, *p* = 0.007), and Early-2 (Z = −3.30, *p* = 0.001), Late-1 (Z = 2.04, *p* = 0.041), and Late-2 (Z = 2.67, *p* = 0.008) (Figure 4F). The remaining comparisons in this study did not differ significantly.

**Figure 5:**
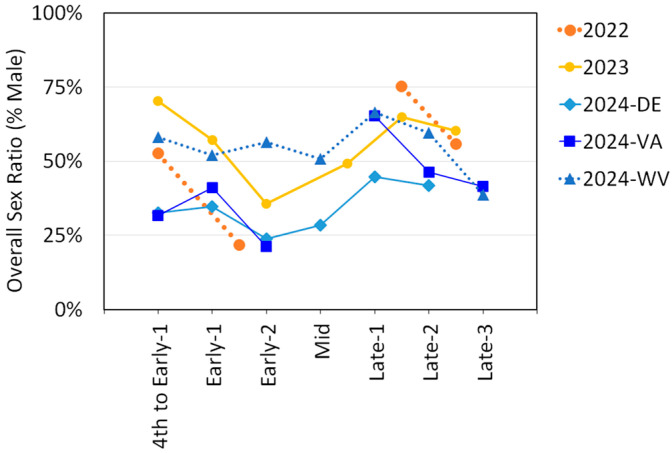
The overall sex ratio (percent males) of *L. delicatula*, spotted lanternflies (SLF), over time for each year of study, with the 2024 data separated by state.

The sex ratios were determined for each trapping period by dividing the number of males by the sum of males and females. Sex ratios for adult SLF for each trapping period for each year and for each of the three states in 2024 are illustrated in Figure 5. All datasets, with the exception of West Virginia in 2024, showed a decrease in sex ratio between Early-1 and Early-2, where the proportion of males dropped 20–34%. All datasets also showed an increase in sex ratio between Early-2 and Late, and another drop in sex ratio as Late progressed.

**Figure 6:**
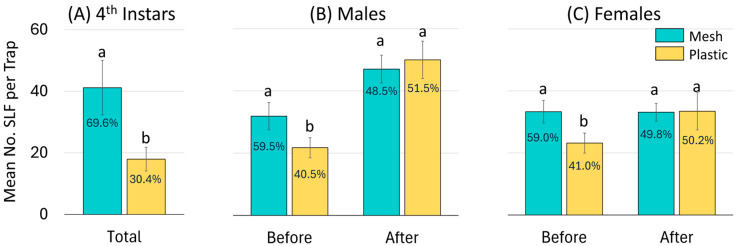
A summary over all three years combined of the mean number of *L. delicatula*, spotted lanternflies (SLF), captured per mesh or plastic trap bag per trapping period for (**A**) 4th instars, and before and after oviposition commenced for (**B**) adult males, and (**C**) adult females. Each bar is labeled with its corresponding frequency. Pairs of bars topped with the same letter are not statistically different (*p* < 0.05).

For all three years combined, an average of 18.0 (±3.8) 4th instars were captured per plastic bag as opposed to 41.2 (±8.9) per mesh bag per trap and trapping period, which was significantly different (Z = −7.38, *p* < 0.0001, N = 286). The overall frequency of 4th instar SLF captured in mesh and plastic bags was 70% and 30%, respectively (Figure 6A).

For all three years combined, a total of 17,061 adults were captured in all trapping periods that ended before the onset of oviposition (21 September), and 21,425 were captured in all trapping periods that ended after that date. Before oviposition started, an average of 21.7 (±3.2) males were caught in each plastic bag, whereas 31.9 (±4.4) males were caught in each mesh bag, which was significantly different (Z = −5.31, *p* < 0.0001, N = 155) (Figure 6B). Also before oviposition, each plastic and mesh bag captured on average 23.2 (±3.0) and 33.2 (±3.7) adult females, respectively, which was significantly different (Z = −6.14, *p* < 0.0001, N = 155) (Figure 6C). In total, mesh and plastic bags captured 59% and 41% of adult SLF, respectively, prior to the onset of oviposition.

After oviposition started, rates of capture were similar for plastic and mesh bags for both males ($\bar{x}$ = 50.0 ± 6.0 and 47.0 ± 4.5, respectively) (Z = −0.44, *p* = 0.660) and females ($\bar{x}$ = 33.4 ± 3.3 and 33.1 ± 2.9, respectively) (Z = −0.86, *p* = 0.390) (Figure 6B,C).

## 4. Discussion

This set of studies tested a modified trapping method based on our current understanding of SLF aggregation behavior and found that keeping SLF alive in a mesh collection bag attached to the tree improved trap efficacy. Additional information was revealed about SLF biology showing that 4th instars strongly responded to aggregations, and that adults responded less to aggregation signals after oviposition was observed. Research to understand and describe how SLF locate conspecifics has been progressing incrementally, with accumulating evidence to suggest that SLF communicate for the purposes of aggregation and mating through multimodal sensory mechanisms, detecting volatile chemicals from their host plants, honeydew, and bodies, as well as substrate vibrations [35,37,39,40].

By facilitating the conveyance of signals from live-captured SLF in mesh collection bags pinned to the tree in the current study, mesh bags significantly and reliably improved trap capture of 4th instars and adult SLF of both sexes prior to mating. Since the main difference between the mesh bag and plastic bag treatments was the presence or absence of SLF-generated signals, these results suggest that signals from captured SLF enhanced trap-entering behavior prior to oviposition time by drawing in more SLF for aggregation and mating.

A very strong preference for traps with mesh collection bags was exhibited in all three years by 4th instar SLF, with mesh bags capturing more than double the 4th instars than plastic bags (Figure 6). Previous work has shown SLF aggregation behavior to occur in all nymphal stages [32,51]. The present study confirmed that aggregation behavior is pronounced in the 4th instar and revealed that 4th instar aggregation is guided by conspecific signals which continue into the Early adult stage. Although field observations of large SLF aggregations typically involve adults, this experiment found that 4th instars were even more strongly attracted to aggregation signals than adults. In the first trapping period in 2024, mesh bags captured nearly four times as many 4th instars than plastic bags. This preference persisted for 4th instars during all trapping periods in which 4ths were present in high numbers.

As SLF progressed through their adult physiological stages, their primary activities also progressed through aggregating, mating, and oviposition, and their preferences for traps with mesh or plastic collection bags correspondingly shifted. Early adults of both sexes showed significant preferences for mesh bags over plastic bags, corresponding with early formations of aggregations while feeding. However, after mating was observed, preferences became less pronounced.

As nymphal stages occur over time as frequency curves with ends that overlap [10,32], so will different adult physiological stages and activities. Some late-developing SLF in the aggregation phase (Early) will coincide with some earlier developing SLF that already started mating (Mid) and subsequently ovipositing (Late). In addition, it has been determined that SLF mate and oviposit multiple times [52,53]. This probably means that Late-1 contains a range of SLF including a few that are late-developing and still virgin, and some SLF that are ovipositing for the first time. Likewise, Late-2 and Late-3 periods likely include a few stragglers that are mating or ovipositing for the first time as well as many SLF that have already mated and oviposited and are ready to mate or oviposit again. Therefore, it is important to note that although the first observation of oviposition marks the start of Late-1, we continue to see courtship, mating, and oviposition for several weeks thereafter.

After oviposition began, results in the three years of study varied. Although significantly more Late adult males and females were captured in mesh bags than in plastic bags during trapping periods in 2022 and 2023, in 2024, significantly more Late-2 adult male SLF were captured in plastic bags than in mesh bags. Similarly, with the onset of oviposition in 2024, adult female SLF were captured in numerically (but not significantly) higher numbers on trees with plastic collection bags. Thus, in Late-1 and Late-2 in 2024, significantly more total adults were captured on trees with plastic bags, suggesting the possibility of avoidance of conspecific signals at that time. It is not clear why SLF behavior late in 2022 and 2023 differed from late in 2024. It may be related to differences in replication, latitude, or environmental conditions such as precipitation between the three years of study. To test for meaningful differences between stimuli when the subject organism has a naturally clumped field distribution as do SLF, blocking and good replication are essential [32,51]. While all three of the current studies had significant variation between blocks, in 2024, we doubled the replicates which improved the robustness of the data.

The changes in SLF behavior seen over time in 2024 mirrored patterns seen in other laboratory and field studies on SLF. For instance, laboratory bioassays found that SLF males were attracted significantly to volatiles extracted from conspecific adults of each sex during Early, and only from females during Mid, but not to either sex during Late [37]. In a set of field studies conducted in 2020 and 2021, in which enclosures on trees containing live adult males or females were used as lures, enclosures containing male SLF captured significantly more male SLF than enclosures containing female SLF from Early-1 to Late-1 [54]. The same study also found that from Late-1 to Late-3, enclosures containing female SLF captured significantly more adult females than enclosures containing male SLF or controls.

Attraction to signals generated by members of the same sex is indicative that those signals carry an aggregation function [55]. SLF honeydew has been shown to attract SLF of the same sex and likely plays an important role in SLF aggregation behavior [39]. Fresh SLF honeydew may signal the presence of both the host plant and SLF that are actively feeding. Honeydew may contain volatile semiochemicals that convey information about the emitter’s sex and physiological state. By testing responses of SLF males and females in a dual choice olfactometer to the headspace volatiles from honeydew, it was found that honeydew excreted by Early males significantly attracted Early males, but not Early females [39]. Conversely, honeydew volatiles from Early females did not attract Early males, but a trend was seen for Early females orienting to Early female honeydew volatiles [39]. Similarly, in a field study comparing SLF attraction to lures of either hexane controls, SLF body extract in hexane, or a combination of SLF body extract plus honeydew, the latter combination significantly attracted males from Mid to Late-2, and females from Late-2 to Late-3, whereas body extract in the absence of honeydew resulted in negative responses by both sexes during Late [36]. Together, the body of evidence from the laboratory and field experiments suggests that volatiles from SLF bodies and honeydew play an important role in the location of conspecifics during aggregation, by mate-seeking males, and by females during oviposition time, whereas extracted body volatiles without honeydew may be a deterrent, particularly after oviposition has begun [36].

In 2024, with trapping starting earlier in the season and farther south, the effects of trap bags could also be evaluated for 3rd instars. Mesh bags captured significantly fewer 3rd instars than plastic bags, which is not surprising because 3rd instars could escape through the holes in the mesh.

Adult SLF sex ratios in this study generally aligned with previously documented patterns of SLF sex ratio progression over time, whereby a shift towards a female-skewed sex ratio is observed roughly two weeks prior to mating (Early-2), then reverts when mating begins (Mid), and then becomes more male-skewed after oviposition ensues (Figure 5) [54,56,57,58,59]. This phenomenon has been well-documented, but how or why it occurs is still not fully understood.

This study is limited in that it does not inform us about the active space of the modified trap, i.e., the distance from which the SLF producing signals inside the mesh bags can be detected by and attract free-living SLF [60,61,62]. Attraction over short distances of both fourth instar and adult SLF to synthetic low amplitude substrate vibrations in the range of 60 Hz has been demonstrated in the laboratory [40]. The active space of insect substrate vibrations generally is confined to the substrate on which the vibration is generated, and attenuates after several meters [60], but can transfer through air short distances between plants [63]. However, the active space of airborne pheromones travelling downwind is much greater, potentially attracting insects from ten to over 100 m away [64,65,66]. Although fourth instar SLF are limited to walking and hopping, SLF semiochemicals released through the mesh bags may have increased the numbers of fourth instars that arrived from neighboring trees, and both the vibratory component and chemical component may have increased the number that arrived near trap entrances as well as trap entry rates. Adult SLF were observed flying to and landing near the mesh traps (MFC, pers. obs.). Since adult SLF are capable of flight, improved trap performance for adults may have resulted from a combination of higher arrival rates from greater distances and improved trap entry rates. As with SLF, a study on live-trapped brown marmorated stink bugs, *Halyomorpha halys* (Stål, 1855) (Hemiptera: Pentatomidae), also showed improved trap capture compared to lethal traps [43]. Similarly, a study on bimodal trapping for the brown marmorated stink bug found that adding female vibrational signals to pheromone traps significantly and synergistically improved trap entry rates of both sexes [67,68]. More research is needed to address the many remaining questions about SLF biology and behavior and to further develop improved traps and lures for SLF.

## 5. Conclusions

We compared SLF circle traps equipped with the standard plastic collection bags, that quickly kill captured SLF, with SLF circle traps equipped with mesh collection bags attached to the tree, which live-trapped SLF and allowed them to feed and generate signals on the tree trunk from inside the bag. Traps with mesh collection bags captured significantly more 4th instar and adult SLF than traps with plastic collection bags prior to oviposition in three years of field trials.

## Figures and Tables

**Table 1:** The experimental design showing trapping dates for each trapping period, trap rotations, stages targeted, and number of block replicates.

Dates ^a,b^	Rotation	Trapping Period	Block Samples (N)
2022 ^a^			
8/2–8/16	1	4th to Early-1	13
8/16–8/30	2	Early-1 to Early-2	13
9/13–9/27	3 ^c^	Mid to Late-1	13
9/27–10/12	4	Late-1 to Late-2	13
10/12–10/26	5	Late-2 to Late-3	13
2023 ^a^			
7/24–8/14	1	4th to Early-1	13
8/14–8/28	2	Early-1	14
8/28–9/11	3	Early-2	14
9/11–9/25	4	Mid to Late-1	14
9/25–10/10	5	Late-1 to Late-2	14
10/10–10/25	6	Late-2 to Late-3	13
2024 ^b^			
7/17–8/6	1	4th to Early-1	26
8/7–8/23	2	Early-1	32
8/24–9/8	3	Early-2	23
9/9–9/20	4	Mid	20
9/21–10/4	5	Late-1	26
10/5–10/18	6	Late-2	20
10/19–11/6	7	Late-3	19

^a^ Date ranges in 2022 and 2023 indicate when traps were set to when trap bags were collected. ^b^ Date ranges in 2024 indicate a range of time in which trap bags were collected, since traps in different blocks were serviced by five different teams in different states and counties and were not all set and collected at the same time. ^c^ This round was counted in the field rather than in the laboratory and sex was not determined.

**Table 2:** The total number and frequency (%) of SLF observed, through 26 pairs of collection bags, to be alive and in a feeding posture at the ends of two trapping periods in 2022.

Stage	Treatment	Alive (%)	Feeding Posture (%)	Total SLF (Dead + Alive)
4th instar				
	Mesh	4 (29%)	1 (7%)	14
	Plastic	1 (6%)	-	17
Adult				
	Mesh	708 (45%)	375 (24%)	1558
	Plastic	6 (1%)	-	521

## Data Availability

The raw data supporting the conclusions of this article will be made available by the authors, without undue reservation.

## Supplementary Materials

The following supporting information can be downloaded at: https://www.mdpi.com/article/10.3390/insects16090930/s1, Table S1: List of field sites.
